# Supplementary material for: Supergene regulation of ant social organization: a P haplotype in workers shifts colony ontogeny towards multiple queens
Source: Commun Biol. 2025 Jul 10;8:1035. doi: 10.1038/s42003-025-08438-5 (PMC12246063; doi:10.1038/s42003-025-08438-5)
Supplement: Supplementary file 2 — Supplementary Information [file 42003_2025_8438_MOESM2_ESM.pdf]

Supplemental Material

Supergene regulation of ant social organization: a *P* haplotype in workers shifts colony ontogeny towards multiple queens

Ornela De Gasperin[1,2*], Pierre Blacher[2], Marina Choppin[2] & Michel Chapuisat[2*]

Affiliations:

[1]Red de Ecoetología, Instituto de Ecología A. C., Xalapa, 91073, Veracruz, México

[2]Department of Ecology and Evolution, University of Lausanne, 1015 Lausanne, Switzerland

***Corresponding authors:**

Ornela De Gasperin; ornela.degasperin@inecol.mx

Michel Chapuisat; michel.chapuisat@unil.ch

Running title: Ontogenetic effects of a social supergene

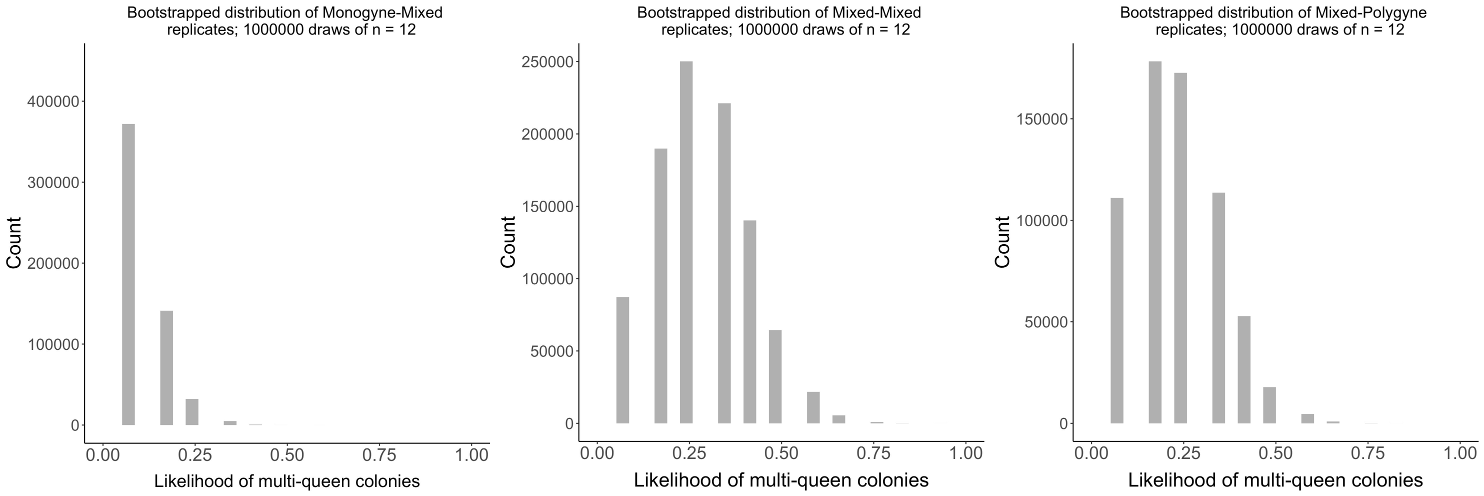

Supplementary Figure 1. Bootstrapped distributions of the 'monogyne-mixed', 'mixed-mixed', and 'mixed-polygyne' treatments (bootstrapped 1000000 times, with replacement, resampling 12 replicates each time).

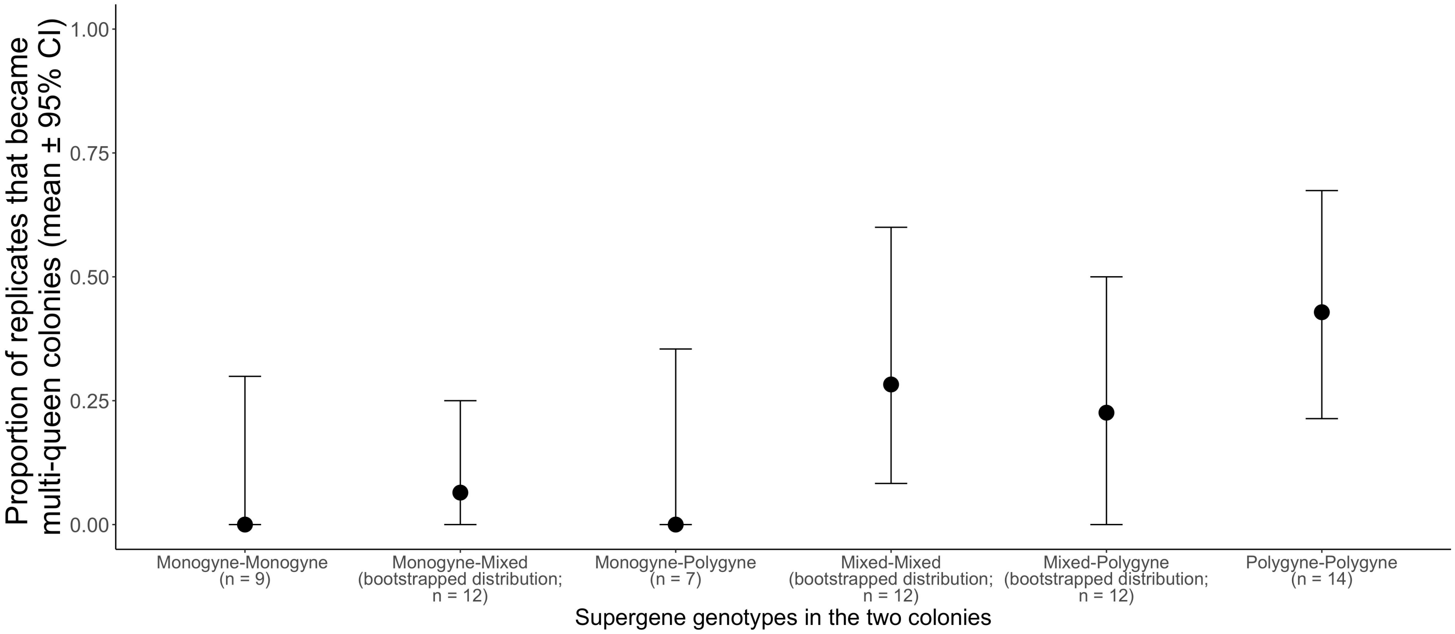

Supplementary Figure 2. Proportions of replicates that became multi-queened. For the ‘monogyne-mixed’, ‘mixed-mixed’, and ‘mixed-polygyne’ treatments, the estimates were calculated from the bootstrapped distributions (bootstrapped 1000000 times, with replacement, resampling 12 replicates each time).
